# Erosive pustular dermatosis: Rapid clearance with nintedanib

**DOI:** 10.1016/j.jdcr.2026.05.054

**Published:** 2026-05-28

**Authors:** Lauren Couture, Paula Atueyi, Victoria Garcia-Albea, Maryanne Senna

**Affiliations:** Department of Dermatology, Lahey Hospital and Medical Center, Burlington, Massachusetts

**Keywords:** antifibrotic therapy, erosive pustular dermatosis, nintedanib, scalp dermatoses, scarring alopecia

## Introduction

Erosive pustular dermatosis (EPD) is a rare, chronic, inflammatory neutrophilic dermatosis characterized by sterile pustules, yellow crusted plaques, and erosions with a predilection for the scalp and legs.^1^^,^^2^ On the scalp, EPD typically arises in hairless areas and causes scarring alopecia.^3^ The etiology is not fully elucidated, though it occurs in areas of actinic damage and local trauma induced by topical medications, surgery, cryotherapy, burns, laser, and radiotherapy. Given its inflammatory nature, EPD is often treated first line with topical corticosteroids, followed by topical calcineurin inhibitors, systemic steroids, and topical and oral antibiotics.^4^ Below we report a case of EPD that was triggered by use of topical compounded fluorouracil 5% and calcipotriene 0.005% cream (5FU+CAL) for actinic keratoses in a patient with comorbid interstitial lung disease (ILD) whose treatment for ILD with nintedanib, a small molecule tyrosine kinase inhibitor (TKi), led to rapid clearance of EPD.

## Case report

**Fig 1:**
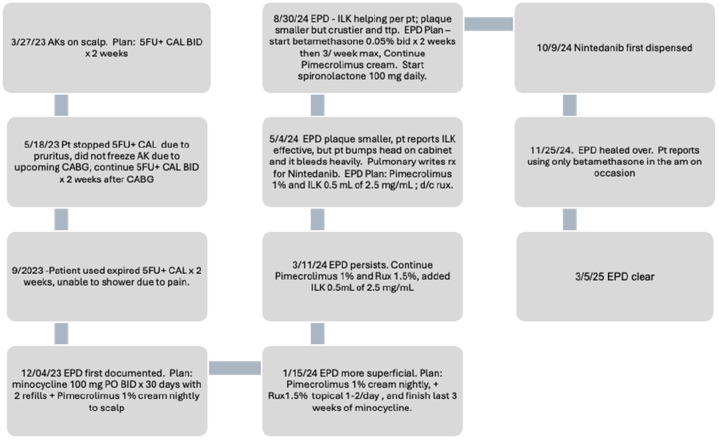
Timeline of EPD Lesion and Treatments. *5FU+CAL*, Compounded combination 5-fluorouracil 5% with calcipotriene 0.005% cream; *Aks*, actinic keratoses; *BID*, twice daily; *EPD*, erosive pustular dermatosis; *ILD*, interstitial lung disease; *ILK*, intralesional Kenalog; *Rux*, ruxolitinib 1.5% topical cream.

**Fig 2:**
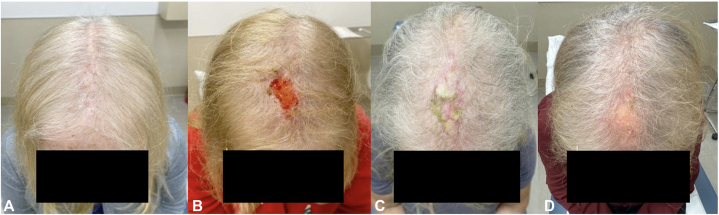
Clinical evolution of EPD from AKs to clearance. **A,** Patient presents on March 10, 2023 with Aks on the scalp. **B,** First presentation of EPD as ulcerative lesion with green, waxy plaques on December 4, 2023. **C,** On August 30, 2024, Patient presents with EPD appearing as non-healing lesion with thick, green crusts on the scalp. **D,** Clearance of EPD with exacerbated alopecia.

In March 2023, a 59-year-old female with a history of Sjogren (treated with hydroxychloroquine), actinic damage, and chronic ILD presented to dermatology for a follow-up for eczematous dermatitis. Actinic keratoses were diagnosed and treated with 5FU+CAL cream, Figs 1 and 2. Early September 2023 (Fig 2), the patient used 5FU+CAL twice daily for 2 weeks resulting in tenderness that prevented the patient from showering due to scalp pain. On examination of the area in December of 2023, a 4 × 2 cm eroded plaque with adherent brown and yellow scale was visualized and debrided to reveal healthy tissue. The patient was diagnosed with EPD and treated with 100 mg minocycline twice daily for 30 days, and 1% pimecrolimus cream to the scalp nightly.

Several treatments were trialed over the next 10 months with incomplete response including betamethasone 0.05% lotion, ruxolitinib 1.5% cream, intralesional triamcinolone, mechanical debridement, and spironolactone 50 mg daily for alopecia secondary to EPD. Intralesional triamcinolone was administered in March and May 2024, but the patient declined ILK at the August 2024 visit and preferred topical treatment. At that visit, she reported worsening yellow crusting, scalp pain, tenderness, and progressive hair loss; examination showed a persistent crusted erosive plaque with scarring alopecia (Fig 2, *C*). Betamethasone was started in August 2024, and Nintedanib was initiated on October 9, 2024 (Fig 1). An October 2024 follow-up visit was canceled, limiting interval assessment on betamethasone alone. By follow-up on November 25, 2024, the affected area was healed, scalp symptoms resolved, and hair regrowth noted; betamethasone was used as needed in the morning (Fig 2, *D*). By March 5, 2025, EPD remained resolved with continued hair regrowth, although crown thinning remained. The patient continued spironolactone 50 mg daily for female pattern hair loss and declined low dose oral minoxidil.

## Discussion

EPD is an auto-inflammatory, neutrophilic folliculitis on a spectrum with pustular pyoderma gangrenosum (PPG) and superficial pyoderma gangrenosum (SPG) that highlight altered wound healing.^1^^,^^5^ At baseline, the dermis is comprised of extracellular matrix (ECM) - mostly collagen, fibroblasts and blood vessels. After disruption by a wound, fibroblasts migrate into the area, a fibrin clot is formed, and fibroblasts remodel the clot with new ECM.^6^ Myofibroblasts are key to organizing the extracellular matrix into scar tissue, but overgrowth of myofibroblasts leads to fibrosis.^6^ Thus, a fibrotic process is likely to play a role in abnormal wound healing.

Based on clinicopathological correlation of 50 EPD cases, Michelerio et al (2019) propose that EPD is caused by superficial, follicular vesiculo-pustules that erode, crust, and granulate. The resulting neutrophilic infiltration causes new epidermis to form above the granulation tissue resulting in a new cycle of vesiculo-pustule formation, erosion, and crusting. This cycle limits wound healing resulting in a chronic wound and scarring alopecia. The latter is caused by excess dermal fibrosis pushing collagen into the deeper reticular dermis.^5^ Reducing fibrosis could alter the disease process allowing for normal wound healing.

Nintedanib, an indoline derivative, exerts anti-fibrotic and anti-inflammatory effects through its action as a triple TKi targeting vascular endothelial growth factor receptor (VEGFR), fibroblast growth factor receptor (FGFR), and platelet-derived growth factor receptor (PDGFR).^7^ We hypothesize that the effects of nintedanib were mediated by a reduction in fibrosis via inhibition of FGFR, though concomitant treatment with topical betamethasone is a potential confounder. Downstream effects of FGFR inhibition include improved collagen deposition in the dermis, promotion of normal granulation, and wound healing by reducing ECM proteins, fibronectin, and collagen 1a.^7^^,^^8^ Due to the side effect profile, 60% of combined patients in TOMORROW and INPULSIS trials reported severe diarrhea and 30% experienced serious adverse events including thromboembolic events and hepatotoxicity.^9^ Oral nintedanib may not be a preferred treatment for EPD. Proposed for the treatment of cicatricial alopecia,^10^ topical nintedanib may be effective for EPD while avoiding the systemic adverse effects. The efficacy of nintedanib may help elucidate the pathophysiology of EPD and encourage the trial of alternative TKis more commonly used in dermatology, such as deucravacitinib, or anti-fibrotic medications used in cicatricial alopecia such as finasteride, dutasteride, PRP, and low-dose metformin for the treatment of EPD.

## Conclusion

EPD remains a therapeutic challenge due to its chronicity, variable response to treatment, and risk of scarring alopecia. The rapid clearance observed after initiation of nintedanib suggests that antifibrotic mechanisms may play a key role in disease modification and highlights the potential use of TKi and other antifibrotic agents to treat this challenging condition. Further controlled studies are needed to clarify the efficacy, safety, and relevance of antifibrotic therapies in the management of EPD.

## Conflicts of interest

None disclosed.
